# Deregulated Lysophosphatidic Acid Metabolism and Signaling in Liver Cancer

**DOI:** 10.3390/cancers11111626

**Published:** 2019-10-23

**Authors:** Eleanna Kaffe, Christiana Magkrioti, Vassilis Aidinis

**Affiliations:** 1 Biomedical Sciences Research Center “Alexander Fleming”, 16672 Athens, Greece; Kaffe@Fleming.gr (E.K.); Magkrioti@Fleming.gr (C.M.); 2 Department of Immunobiology, Yale School of Medicine, New Haven, CT 06519, USA

**Keywords:** Liver fibrosis, cirrhosis, hepatocellular carcinoma (HCC), cholangiocarcinoma, Lysophosphatidic acid (LPA), Autotaxin (ATX; *ENPP2*), LPA receptors (LPARs)

## Abstract

Liver cancer is one of the leading causes of death worldwide due to late diagnosis and scarcity of treatment options. The major risk factor for liver cancer is cirrhosis with the underlying causes of cirrhosis being viral infection (hepatitis B or C), metabolic deregulation (Non-alcoholic fatty liver disease (NAFLD) in the presence of obesity and diabetes), alcohol or cholestatic disorders. Lysophosphatidic acid (LPA) is a bioactive phospholipid with numerous effects, most of them compatible with the hallmarks of cancer (proliferation, migration, invasion, survival, evasion of apoptosis, deregulated metabolism, neoangiogenesis, etc.). Autotaxin (ATX) is the enzyme responsible for the bulk of extracellular LPA production, and together with LPA signaling is involved in chronic inflammatory diseases, fibrosis and cancer. This review discusses the most important findings and the mechanisms related to ATX/LPA/LPAR involvement on metabolic, viral and cholestatic liver disorders and their progression to liver cancer in the context of human patients and mouse models. It focuses on the role of ATX/LPA in NAFLD development and its progression to liver cancer as NAFLD has an increasing incidence which is associated with the increasing incidence of liver cancer. Bearing in mind that adipose tissue accounts for the largest amount of LPA production, many studies have implicated LPA in adipose tissue metabolism and inflammation, liver steatosis, insulin resistance, glucose intolerance and lipogenesis. At the same time, LPA and ATX play crucial roles in fibrotic diseases. Given that hepatocellular carcinoma (HCC) is usually developed on the background of liver fibrosis, therapies that both delay the progression of fibrosis and prevent its development to malignancy would be very promising. Therefore, ATX/LPA signaling appears as an attractive therapeutic target as evidenced by the fact that it is involved in both liver fibrosis progression and liver cancer development.

## 1. Introduction/Lysophosphatidic Acid (LPA)

LPA is a bioactive lipid that belongs to the class of lysophospholipids, which are phospholipids with only one fatty acyl chain. LPA consists of a glycerol backbone, an attached phosphate head group and an ester-linked aliphatic chain (fatty acid) of varying length and degree of saturation depending on the precursor phospholipid. Therefore, it is a group of saturated (14:0, 16:0, 18:0) and unsaturated (16:1, 18:1, 18:2, 20:4, 22:6) acyl species. Moreover, at times the aliphatic chain is ether- (instead of ester-) linked to the glycerol backbone, thus resulting in alkyl or alkenyl analogs of LPA. LPA is present at most biological fluids, such as saliva, urine, cerebrospinal fluid, blood, bronchoalveolar lavage fluid (BALF) and other [1]. Compared to plasma, serum LPA levels are much higher, due to the release of its major precursor, lysophosphatidylcholine (LPC), from activated platelets during coagulation and the subsequent transformation of LPC to LPA [2,3]. Moreover, the levels of LPA are much lower (~0.7 μM) than the levels of LPC (~200 μM) and with a different species distribution (LPA: 18:2 > 20:4 > 18:1; LPC: 16:0 > 18:1/18:0 > 20:4).

Non-coincidentally, LPA is being characterized as a growth factor-like molecule. Indeed, it implements a variety of functions on nearly all cell types, such as acting as a proliferative and pro-survival signal, inducing cellular invasion and migration, stimulating smooth muscle and fibroblast contraction, differentiation, cytoskeletal rearrangement, secretion of cytokines/chemokines and other effects [2]. Therefore, LPA is implicated in myriad processes; some being cancer-related which will be mentioned later in detail, and other processes such as vascular homeostasis [4,5], lymphocyte homing [6,7], development of the nervous system [8], demyelination and neuropathic pain [9], skeletal development and remodeling [10,11] and stem cell physiology [12]. This vast number of actions is enabled and mediated through a plethora of LPA receptors, which are discussed below.

## 2. LPA Metabolism

**Figure 1:**
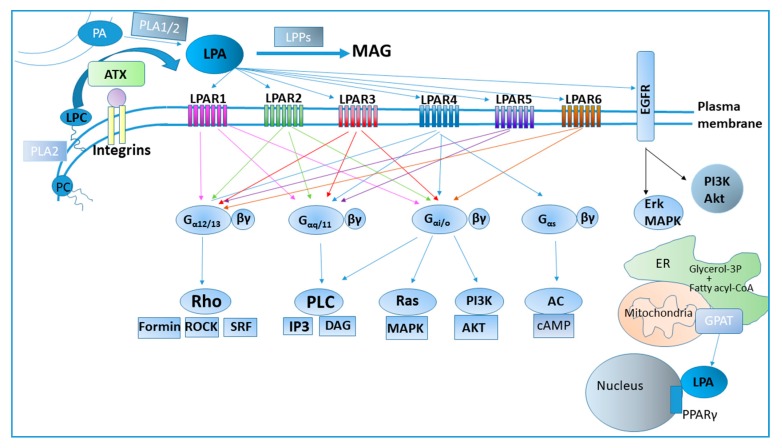
Lysophosphatidic acid (LPA) metabolism and signaling. AC: adenylyl cyclase; AKT: AKR mouse thymoma (albeit the name does not represent its function); ATX: Autotaxin; cAMP: cyclic adenosine monophosphate; DAG: diacylglycerol; EGFR: Epidermal growth factor receptor; ER: endoplasmic reticulum; ERK: extracellular signal-regulated kinase; GPAT: glycerol-3-phosphate acyltransferase; IP3: Inositol trisphosphate; LPAR: lysophosphatidic acid receptor; LPC: l;ysophosphatidylcholine; LPPs: lipid phosphate phosphatases; MAG: monoacylglycerol; MAPK: Mitogen activated protein kinase; PA: phosphatidic acid; PI3K: Phosphoinositide 3-kinase; PLA: phospholipase A; PLC: Phospholipase C; PPARγ: Peroxisome Proliferator-Activated Receptor gamma; Rho: Ras homolog gene; ROCK: Rho-associated, coiled-coil-containing protein kinase; SRF: serum response factor.

The main precursor of LPA is LPC, while other lysophospholipids such as lysophosphatidylethanolamine and lysophosphatidylserine can also serve as LPA precursors. LPC is synthesized by phospholipases A_2_ (PLA_2_) employing membrane or extracellular phosphatidylcholine (PC) as a substrate, while, simultaneously, free fatty acids (FFA) are also produced (Figure 1). LPC is abundant in plasma, where it is found associated with albumin or oxidized low-density lipoprotein (oxLDL) [2,13]. The enzyme responsible for the extracellular hydrolysis of LPC, and the other lysophospholipids, to LPA is Autotaxin (ATX), a secreted glycoprotein with lysophospholipase D (lysoPLD) activity [14]. Although ATX belongs to the ectonucleotidepyrophosphatase-phosphodiesterase (ENPP) protein family [15], ATX distinguishes from the other family members by being secreted (contrary to the membrane ENPPs) [16] and by possessing lysoPLD properties. In fact, most of the functions attributed to ATX are mediated through its product, LPA [2,3,17]. Moreover, ATX has been suggested to bind to membrane integrins [18,19,20] possibly bringing LPA into proximity with its receptors and, thus, localizing LPA effects. ATX consists of two N-terminal somatomedin B-like domains (SMB1 and SMB2), a central phosphodiesterase (PDE) domain encompassing its catalytic site along with a deep hydrophobic pocket that accommodates the acyl chain of its substrate, and a C-terminal nuclease-like domain (NUC) [2,13], whereas its crystal structure has been solved allowing apprehension of its function [19,21,22]. The catalytic activity of ATX is largely affected by the length and saturation degree of the fatty acid moiety on its substrate with the preference order being 18:0 << 16:0 < 14:0 < 12:0 and 18:0 << 18:1 < 18:3 [14,21,23].

Complete genetic deletion of the ATX gene (*Enpp2*) in mice is embryonically lethal due to aberrant vascular and neuronal development [8,24], although ATX is dispensable for adult life, as inducible complete genetic deletion of *Enpp2* in adult mice is viable [25]. In adults, ATX is expressed in several tissues with the most prominent being the adipose tissue, the central nervous system (CNS) and the reproductive organs. In fact, ATX derived from the adipose tissue is secreted in the plasma and accounts for the 38–50% of plasma LPA [26,27]. Thus, ATX is the key responsible enzyme for the bulk amount of plasma LPA as further evidenced by the fact that genetic deletion or pharmacological inhibition of ATX inhibits systemic LPA levels by 80–90% [25]. Notably, ATX expression has been shown to be induced by several proinflammatory factors (lipopolysaccharide, tumor necrosis factor (TNF), interleukin 6 (IL-6), galectin-3) [2,28], hence linking it with inflammatory conditions. Additionally, LPA has been suggested to downregulate ATX expression, in the absence of inflammatory factors [29].

Apart from ATX, other possible LPA synthetic pathways also exist [1], such as LPA generation from phosphatidic acid (PA) (Figure 1). Phospholipids or diacylglycerol are first transformed into PA and the latter is deacylated by phospholipases A_1_ or A_2_ [30]. Secretory PLA_2_ has been found to produce LPA from PA in a system of erythrocyte microvesicles, whereas secretory and cytoplasmic PLA_2_s can produce LPA in ovarian cancer cell cultures [31,32]. On the other hand, two membrane-bound PA-specific PLA_1_ enzymes, mPA-PLA_1_α and mPA-PLA_1_β, can produce 2-acyl-LPA when overexpressed in insect cells [33]. Nevertheless, the importance of LPA production via the PLA-mediated pathways in vivo has not been proven nor is it established as is the ATX-mediated LPA production. Finally, LPA is an intermediate metabolite in de novo lipogenesis (DNL), both in adipose tissue and in liver. In this pathway, LPA is generated upon the acylation of glycerol-3-phosphate by glycerol-3-phosphate acyltransferase (GPAT) using acyl-CoA as a lipid donor (Figure 1) [34]. All 4 GPAT isoforms are associated with intracellular organelles (mitochondria or endoplasmic reticulum), therefore any LPA generated through this pathway will be intracellular. Interestingly, GPAT1 is primarily located in the mitochondria of hepatic cells ([34] and references therein).

Τhe catabolism of LPA occurs through lipid phosphate phosphatases (LPPs), three proteins (LPP1–3) that are located on the plasma membrane, with their active site being extracellular and thus able to catabolize extracellular LPA into monoacylgycerol (MAG) [17,35]. Mice with hypomorphic *Lpp1* show increased LPA concentration in plasma and a longer half-life of LPA [36]. Moreover, other enzymes like phospholipases and LPA acyltransferases can also metabolize LPA [1]. Furthermore, liver is a major organ for LPA clearance, as shown by detection of exogenously administered LPA in the liver [35].

## 3. LPA Receptors and Signaling

LPA signals through many receptors that exhibit a widespread, but differential, cell and tissue distribution, and overlapping specificities (Figure 1). Lysophosphatidic acid receptor 1 (LPAR1) was the first receptor identified with a high affinity for LPA in 1996 [37]. Both LPAR1 and LPAR2 couple with G_αi/o_, G_αq_ and G_α12/13_ ([38] and references therein). An orphan G protein-coupled receptor (GPCR) was later designated LPAR3, which couples with G_αi/o_, G_α12/13_ and G_αq_ [38,39]. LPAR1–3 are phylogenetically related and have been shown to have a preference for acyl-LPAs compared to their alkyl/alkenyl LPA analogs [40]. Another orphan GPCR, purinergic receptor 9/ G protein coupled receptor 23 (p2y9/GPR23), was later identified as the fourth LPA receptor (LPAR4), albeit phylogenetically distant from the Edg family, therefore deriving from a separate ancestor sequence [41]. LPAR4 has been found to transduce signaling through G_α12/13_-Rho kinase, G_αq_ and calcium mobilization or G_αs_ and cyclic adenosine monophosphate (cAMP) influx [42]. Orphan GPCR, GPR92, was identified as LPAR5, mediating the LPA signaling through G_α12/13_ and G_αq_ [43], whereas orphan GPCR p2y5 was identified as LPAR6 transducing signaling through G_αi_ and G_α12/13_ [44,45]. Cluster of differentiation 97 (CD97), another GPCR, has been found to heterodimerize with LPAR1, thus enhancing LPA signaling through Rho kinase [46]. GPCR GPR87 and GPCR P2Y (10) have also been proposed as LPA receptors [47,48]. Out of the numerous G_α_ proteins that couple with GPCRs, G_αi_ induces the mitogenic pathway Mitogen-activated protein kinase (MAPK), Ras-Raf-MEK-ERK, and the pro-survival Phosphoinositide 3-kinase (PI3K)/Akt pathway, G_α12/13_ stimulates Ras homolog gene A (RhoA)-dependent cytoskeletal remodeling, cell migration and invasion and G_αq_ stimulates phospholipase C [1,2,49,50]. In addition, LPA may lead to changes in cAMP levels via G_αi_, G_αs_ or G(_βγ_) subunits.

Noteworthy, nuclear receptor Peroxisome Proliferator-Activated Receptor gamma (PPARγ) has also been proposed as an intracellular receptor for LPA [51], with alkyl- analogs of LPA being more potent agonists for PPARγ compared to the more common acyl-LPA species [52]. Moreover, LPA is capable of transactivating tyrosine kinase receptor EGFR inducing multiple pathways [53,54,55,56]. It becomes apparent that the LPAR spatiotemporal and cell-specific expression profile will affect the final LPA effects at any given circumstance. Both the differentiated LPAR expression patterns [38] and their ability to induce various signaling pathways and secondary messengers, allow LPA to have a great diversity of physiological and pathological effects. This plethora of LPA effects is also affected by the local concentration of LPA, the species of LPA and their affinity to LPARs, as well as the presence of potential LPAR agonists and antagonists or other growth factors that synergize with LPA.

## 4. LPA in Chronic Inflammation

Many studies have implicated LPA signaling in chronic inflammation. LPA promotes inflammation by affecting the endothelium in several ways such as stimulating endothelial cell migration [57,58], secretion of chemokines/cytokines [59,60,61] or expression of cell adhesion molecules [62] and regulating endothelial barrier integrity [63,64]. Moreover, LPA signaling is crucial for the homing of T lymphocytes in lymph nodes as it promotes transmigration of circulatory T lymphocytes into lymph nodes by stimulating the motility and permeability of high endothelial venules (HEVs) [65] and/or by inducing chemokinesis of T lymphocytes [66].

As mentioned earlier, a high percentage of plasma LPA is attributed to the adipose tissue, through adipose-specific ATX expression and secretion [26,27]. In diet-induced experimental obesity, LPA and ATX affect adiposity, albeit in a controversial manner [26,27,67,68]. Given the predominant role of adipose tissue in obesity and the fact that obesity, by altering the adipose secretome, activates the innate immune system and triggers low-grade chronic inflammation [69], a connection between LPA and chronic inflammation can be foreseen. In a recent study, adipose tissue-specific deletion of *Enpp2* (ATX gene) in High-Fat diet (HFD)-fed mice decreased adipocyte size and adipose tissue inflammation locally [68], which is related to less chronic low-grade inflammation.

The implication of LPA signaling in chronic inflammation has been illustrated in numerous chronic inflammatory diseases, such as idiopathic pulmonary fibrosis (IPF), a chronic, interstitial lung disease caused by aberrant wound healing. Increased levels of LPA in the BALF [64] and increased ATX staining in the lungs have been detected in IPF patients [70]. Moreover, ubiquitous genetic deletion of *Lpar1* or *Lpar2* and conditional deletion of *Enpp2* from bronchial epithelial cells or macrophages attenuated pulmonary fibrosis, vascular leak and inflammation, attributed to blocked LPA signaling or decreased LPA levels, respectively [64,70,71]. Apart from regulating the vascular leak, LPA has been found to induce the secretion of IL-8, stimulating neutrophilic influx [72,73], and to activate monocytes into macrophages [74], thus enhancing inflammation. Additionally, LPA signaling is implicated in the fibrotic component of the disease by activating the cornerstone of fibrosis development, Transforming growth factor-β (TGF-β) [75] and by inducing chemotaxis and survival of fibroblasts [64,76]. Of note, an ATX inhibitor, GLPG1690, is in phase III clinical trials (ISABELA 1 and 2; NCT03711162; NCT03733444) and an LPAR1 inhibitor, BMS-986020, is in phase II clinical trials for the treatment of IPF [77,78].

Rheumatoid arthritis (RA) is another chronic, but also autoimmune, disease where deregulated LPA signaling has been detected. Increased LPAR1 and/or LPAR2 levels are detected in the synovium of RA patients compared to osteoarthritis patients [79,80]. Genetic deletion of *Lpar1* completely abolished the disease and pharmacologic inhibition of LPAR1 reduced disease severity, inflammation and bone erosion [80,81]. Similarly, increased ATX levels are detected in patient synovial fibroblasts and local genetic deletion of *Enpp2* in synovial fibroblasts abrogates the disease in modeled RA [82]. TNFα, the key player and current therapeutic target in RA, stimulates ATX expression in synovial fibroblasts in vitro [82] and LPA induces proliferation, migration and secretion of proinflammatory cytokines IL-6 and IL-8 in synovial fibroblasts, providing mechanistic insights for the implication of ATX/LPA/LPAR signaling in RA [82,83].

Atherosclerosis, the main contributor to coronary and peripheral artery disease, is another chronic inflammatory condition where LPA signaling has a prominent role, with *Ppap2b*, the gene encoding an LPA-catabolizing protein, being the most prominent novel susceptibility gene for coronary artery disease (CAD) [84]. Serum LPA levels are raised in patients with acute myocardial infarction (AMI) after the incident [85]. Infarct-related coronary arteries contain higher levels of LPA compared to systemic arterial circulation [86] and the same applies to atheromatous plaques compared to normal arterial tissue [87]. Gradual accumulation of LPA in atheromatous plaques has been shown in experimental atherogenesis [88], with the most probable mechanism involving LPC production during low-density lipoproteins (LDL) oxidation in the plaques and its subsequent transformation to LPA by ATX [59]. LPA has numerous effects on plaque formation and progression, such as induction of monocyte adhesion [59] and oxidized LDL engulfment from macrophages [89], with LPAR4 being the crucial receptor [90]. Other chronic inflammatory conditions where LPA is implicated in are calcific aortic valve disease [91], multiple sclerosis [92], Alzheimer’s disease [93], kidney fibrosis [94,95] and colitis [96]. Hence, ATX and LPA signaling have attracted the interest of researchers and pharmaceuticals as drug targets in the context of the aforementioned conditions.

## 5. LPA Axis in Cancer

The first indication that LPA is implicated in cancer was its ability to activate and induce the proliferation of ovarian and breast cancer cells [97]. ATX was also associated with cancer very early on after its discovery, as it was identified as a motility-stimulating factor in the conditioned medium of melanoma cells [98]. Since then, aberrant LPA signaling has been observed in multiple types of cancer. Increased LPA levels have been found in hepatocellular carcinoma (HCC), non-small cell lung cancer (NSCLC), ovarian carcinoma, pancreatic cancer, thyroid cancer, multiple myeloma and follicular lymphoma [2,99] whereas increased ATX levels have been detected in glioblastoma multiforme, melanoma, breast cancer, HCC, NSCLC, renal cell carcinoma, thyroid cancer, ovarian cancer, pancreatic cancer and several lymphomas [2]. Intriguingly, the ATX encoding gene, *ENPP2,* resides in the human chromosomal region 8q24, a region that contains potential susceptibility loci for different types of cancer [100]. Enhanced LPA signaling through LPARs has also been observed in several types of cancer. Given the fact that LPARs are normally expressed at low levels, their high expression in some cancer entities indicates that they may promote carcinogenesis [101]. Indeed, LPAR1 is crucial in bone metastasis of breast carcinoma, LPAR2 promotes colorectal cancer [102] and aggressiveness of ovarian cancer cells, whereas LPAR3 enhances migration of hepatoma cells ([103] and refer therein). Moreover, increased expression of LPAR3 and ATX in breast cancer biopsies is associated with tumor aggressiveness [104]. On the other hand, LPARs have also been suggested to have inhibitory effects on various cancer types, [103,105,106]. Hence, different LPAR expression profiles in each type of cancer will transduce LPA signaling in a distinct manner producing different outcomes.

Intriguingly, multiple LPA effects are identical with some of the cancer hallmarks, biological capabilities acquired by cancer cells during the progress of carcinogenesis, as have been defined in an attempt to rationalize cancer entanglement [107]. Such effects are survival, proliferation, invasiveness, inflammation, angiogenesis, evasion of growth suppressors and energetic alterations.

Consistent with the carcinogenic hallmark of invasion and metastasis, LPA promotes invasiveness through the GTPase RhoA, a protein involved in actin cytoskeleton organization, and Rho-associated coiled-coil forming protein kinase (ROCK), a known mediator of cell migration [46,108]. It also promotes invasiveness in other ways such as induction of Ras-protein kinase C (PKCA) and Nuclear Factor Kappa B (NFkB), enhancement of Epithelial to mesenchymal transition (EMT) and induction of matrix metalloproteases (MMPs) which proteolytically cleave extracellular matrix (ECM), among others [2,108,109]. Recent research corroborates the LPA-mediated induction of invasion and metastasis through LPAR1, Epidermal growth factor receptor (EGFR) transactivation and invadopodia formation in various cancer cell lines [110], through LPAR2 and Notch signaling in gastric cancer cells [111], or through LPAR2 in osteosarcoma cells [112]. According to another research group, in the case of melanoma, LPA, either produced from the stroma or from inflammatory cells surrounding the tumor, is degraded by cancer cells via LPP3 creating a gradient of LPA (low in the tumor, high in the surrounding area) which drives their outward migration in vivo and in vitro [113,114]. Similarly, ATX levels were found higher in invasive tumor cells compared to tumor core cells in gliobastomas [109]. Moreover, LPA aids cancer cells in adapting to the hypoxic environment through induction of Vascular endothelial growth factor (VEGF), its receptor (VEGFR2) and Hypoxia-Inducible-Factor-1-Subunit-Alpha (Hif-1α) ([101] and references therein) and tightens endothelial cell-cell contact promoting capillary network formation [115], thus satisfying the cancer hallmark of angiogenesis.

Tumor-promoting inflammation is another key cancer hallmark where ATX/LPA are also involved. This was demonstrated in the context of breast cancer, where ATX is mainly produced by adipose tissue adjacent to the tumor rather than the tumor per se, and, through LPA production, promotes the secretion of inflammatory mediators from tumor cells. On the other hand, inflammatory mediators from the tumor cells induce the production of ATX from adipocytes, thus forming a vicious circle which favors tumor-promoting inflammation and cancer progression [116]. A reciprocal connection between inflammatory mediators and ATX levels has been shown in the formation of malignant thyroid tumors, as well [99]. Interestingly, tumor-associated macrophages have also been designated as LPA producing cells, as shown in a recent publication pertaining to ovarian cancer [117]. Therefore, inflammatory mediators induce LPA production which subsequently reinforces the inflammatory response, thus creating a vicious circle that may sustain.

Deregulated cellular metabolism favors also cancer growth. LPA has been shown to induce a metabolic shift towards glycolysis, a key metabolic pathway that fuels cancer cells. In particular, LPA derived from ovarian cancer cells was recently shown to promote a glycolytic shift in ovarian cancer cells [118] and in normal fibroblasts through HIF-1a, thus leading to the transformation of fibroblasts into cancer-associated-fibroblasts (CAFs) [119]. Hence, LPA upregulates the levels of HIF-1α which is considered to be the master regulator of glycolysis in tumors [120]. Moreover, researchers have shown that pancreatic stellate cells undergo lipid remodeling upon their activation into CAFs during carcinogenesis, enabling them to secrete lipids that can support the metabolism and growth of adjacent pancreatic ductal adenocarcinoma cells (PDACs) even at conditions of nutrient deficiency, such as in cancer [121]. A major class of the secreted lipids is lysophospholipids, which can either be uptaken by PDACs and transformed into phospholipids for further membrane synthesis or can be hydrolyzed by ATX giving rise to LPA, which in turn induces proliferation and migration of PDACs. Therefore, LPA in an autocrine or paracrine way is able to deregulate cellular metabolism in both stromal and cancer cells, thus favoring cancer growth.

LPA signaling is also linked to chemoresistance which is related to another cancer hallmark (“evasion of apoptosis and growth suppressors”). LPA inhibits taxol-induced apoptosis in melanoma and breast cancer cells [122] and cisplatin-induced apoptosis in ovarian cancer cells through LPAR2 [123]. LPA has been shown to induce chemoresistance in breast, lung, liver, and thyroid cancer cell lines by inducing the stabilization and nuclear localization of transcription factor Nuclear-factor-erythroid-2-related-factor-2 (Nrf2), which upregulates genes encoding multidrug-resistant transporters and antioxidant proteins [124]. More importantly, inhibition of ATX-mediated LPA production enhances the anticarcinogenic and antimetastatic activity of doxorubicin in an orthotopic breast cancer model [124]. Additionally, increased ATX expression is observed in the tumor vasculature of human renal cell carcinomas upon treatment with sunitinib and the ATX/LPA signaling is thought partly responsible for the acquired resistance to sunitnib [125]. Nonetheless, indications that LPA, through LPAR5, promotes cancer cell apoptosis upon the use of chemotherapeutic drugs, also exist [105].

**Figure 2:**
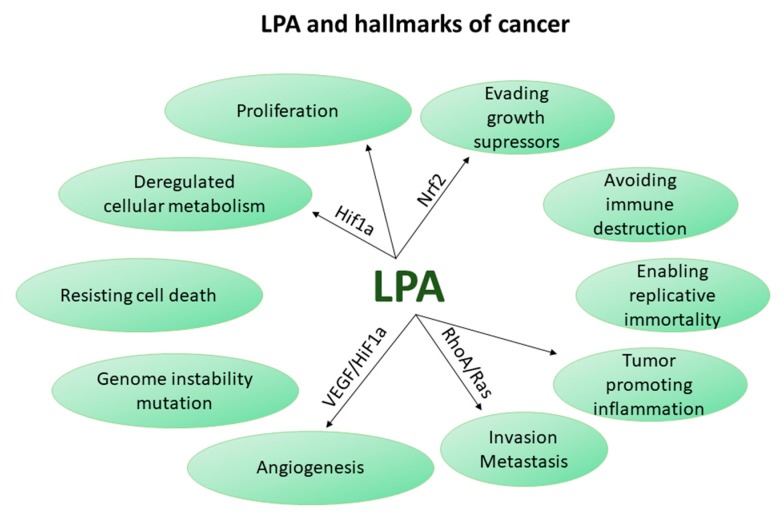
Hallmarks of cancer, as presented in Reference [107], and their relation to LPA. VEGF: vascular endothelial growth factor; HiF-1α: Hypoxia Inducible Factor 1 Subunit Alpha; RhoA: Ras homolog gene A; Nrf2: Nuclear factor erythroid 2-related factor 2.

All the above studies indicate that LPA may have a strong carcinogenic role across several types of cancer via modulating vital cellular processes as summarized in Figure 2. This concept is further supported by studies with genetic deletion or overexpression of ATX and/or LPARs in vivo. Expression of human ATX, LPAR1, LPAR2 or LPAR3 in transgenic mice is sufficient to induce late-onset mammary carcinomas [126]. Angiogenesis, tumor growth, and metastasis of cancer cells are promoted by ATX through galectin-3 in an orthotopic melanoma model [127]. Furthermore, deletion of *Lpar2* suppresses tumorigenesis in a model of colitis-associated cancer [102]. Moreover, ATX genetic deletion in PDACs or pharmacologic inhibition in an orthotopic model of PDAC led to a decrease in tumor growth [121]. Therefore, targeting the LPA axis, either at the level of LPA production by ATX or at the level of LPA signaling through its receptors, seems promising in the context of cancer therapeutics. Apart from breast, colorectal, melanoma and pancreatic cancer, the carcinogenic role of ATX/LPA was also evidenced in the context of liver cancer as discussed in detail below.

## 6. Liver Cancer

The liver is a metabolic organ as evidenced by the fact that all liver cell types have metabolic functions. The metabolic functions of hepatocytes (Heps) determine the immune/metabolic functions of the non-parenchymal liver cells (NPCs; immune cells, endothelial cells, hepatic stellate cells) and vice versa. Heps can be conceived of as the orchestrators and NPCs as the gatekeepers of liver functions. NPCs sense damaging insults and, by secreting several factors, induce Hep proliferation which replenishes their population. Once the damaging insult is cleared from the liver, signals from Heps and, probably, NPCs terminate the immune responses. This crosstalk allows the human liver to retain its homeostasis even if it is daily exposed to a multitude of damaging insults (dietary, microbial, toxins and drugs) [128]. However, extensive exposure to insults (i.e., fat, sugar, alcohol, viruses or autoimmune agents) may damage Heps or cholangiocytes evoking an uncontrolled activation of NPCs that leads to chronic liver disease. The unresolved wound healing response progresses to liver cirrhosis and liver cancer, a deadly complication of chronic liver diseases [129]. The only available therapy for liver cirrhosis is liver transplantation, whereas no effective therapy exists for liver cancer. In fact, liver cancer accounts for 80% of liver disease- related deaths [130].

The most prevalent type of liver cancer is HCC which accounts for 90% of liver cancers, with cholangiocarcinoma (CCA) following. HCC is one of the leading causes of cancer-related deaths globally [131,132]. Its high mortality is owed to late diagnosis [130] and scarcity of treatment options. Treatments such as tumor resection, radiofrequency ablation and liver transplantation can only be applied to a few patients, whereas the only available drug for the treatment of unresectable HCC, a multi-kinase inhibitor under the name of sorafenib, only extents survival for two to three months [130,133]. The major risk factor for HCC is liver cirrhosis while the underlying cause of liver cirrhosis is also significant. The dominant risk factors for cirrhosis-related HCC are viral hepatitis in Asia and Africa and Alcoholic liver disease (ALD) and Non-Alcoholic Fatty Liver disease (NAFLD) coexisting with Obesity and Type 2 Diabetes mellitus (T2DM) in western countries [130].

CCAs are a group of malignancies of the biliary epithelium (cholangiocytes) comprising invasive carcinomas that arise in the intrahepatic, perihilar or extrahepatic biliary tree [134]. CCA, that accounts for 13% of cancer-related deaths [135,136], is highly aggressive and carries a dismal prognosis (median survival less than one year), due to early metastatic dissemination to lymph nodes, striking resistance to conventional chemotherapy and high rate of recurrence after curative resection [137,138]. CCA is characterized by a dense tumor reactive stroma (TRS) which promotes its progression by secreting several factors [137,139,140]. Chronic cholangiopathies such as Primary Sclerosis Cholangitis (PSC) [141] and Caroli Disease are established risk factors for CCA development suggesting that their inflammatory and fibrotic component provides a tumor-promoting microenvironment [142]. Several lifestyle-related parameters causing chronic hepatic inflammation and cholestasis are also important risk factors [143]. Similarly to HCC, metabolic syndrome, obesity, T2DM, NAFLD, alcohol abuse, viral hepatitis and cirrhosis are risk factors for CCA as well [136,144].

## 7. Deregulated LPA Metabolism and Risk Factors for Liver Cancer

The extracellular LPA metabolism encompasses enzymes involved in LPA production (ATX) or degradation (LPPs). Most of the available in vivo/in vitro studies pertaining to chronic liver diseases, HCC and the LPA axis refer to ATX rather than LPPs. Therefore, this review mostly focuses on the role of ATX in HCC, however the few studies addressing the levels of LPPs in these pathological conditions will be also discussed.

### 7.1. Cirrhosis-Related Liver Cancer

The major risk factor for HCC and CCA is liver cirrhosis suggesting that aberrant myofibroblast proliferation and ECM deposition support the malignant transformation of damaged Heps or cholangiocytes. Liver cirrhosis develops upon chronic liver damage of different etiologies (viral, autoimmune, metabolic, alcohol, cholestatic). ATX levels positively correlated with liver fibrosis stage and serve as a predictor of liver disease severity and overall survival regardless of the liver disease etiology [145,146,147,148,149], whereas serum LPA levels correlated with ATX levels [147]. Moreover, HCC specimens with underlying cirrhosis display higher ATX expression levels compared to non-cirrhotic HCC specimens [150]. These results provide a strong indication for the association of serum ATX/LPA levels with liver cirrhosis. Notably, ATX expression was detected in both tumoral and non-tumoral areas in cirrhotic patients with HCC suggesting that both Heps and NPCs are sources of ATX in HCC [151]. On the contrary, in a normal liver or upon chronic liver damage, ATX is mainly produced by Heps rather than NPCs [152].

However, the causal relationship between ATX/LPA and cirrhosis could have an opposite direction, too, as LPA produced by ATX exerts a pathogenic role in hepatic stellate cells (HSCs) activation [152], the key driver of liver cirrhosis. During chronic liver damage, the signals secreted from damaged epithelial cells are sensed by the NPCs leading to their activation. Bioactive lipids appear as good candidates for this crosstalk. This was explicitly shown for the ATX/LPA axis. In a normal liver, ATX is mainly produced by Heps at low levels rather than NPCs [152] whereas LPARs (LPAR1, LPAR3 and LPAR6) are expressed mainly in NPCs rather than Heps [153] suggesting that LPA derived via ATX from Heps acts in a paracrine way to adjacent NPCs. Similarly, in chronic Carbon tetrachloride (CCl4)-induced liver fibrosis, CCl4-damaged Heps induce the activation of PLA_2_ which hydrolyzes PC to LPC, and, subsequently, the increased LPC levels induce ATX expression that hydrolyzes LPC to LPA [152]. LPA secreted by Heps stimulates HSCs activation. Inhibition of LPA-mediated Heps-HSC crosstalk via genetic deletion of *Enpp2* in Heps halted disease progression in that model [152]; in particular, circulatory and hepatic LPA along with liver fibrosis, infiltration of CD68^+^ macrophages, Gr-1^+^ monocytes and liver lipid deposition were decreased. Hep-specific ATX overexpression only increased liver fibrosis indicating that the major effect of altered LPA metabolism is on the HSC activation. Indeed, LPA was shown to stimulate Alpha-smooth muscle actin (a-SMA) expression and actin rearrangement, proliferation and migration, while inhibit apoptosis in HSCs in vitro, hence promoting their activation and transformation into myofibroblasts [152,154]. These results indicate a key role for ATX and LPA in promoting specifically HSC activation in the liver disease progression. Therefore, targeting ATX/LPA signaling may halt the progression of chronic liver diseases to cirrhosis and subsequently to HCC. Indeed, Hep-specific ATX deletion shows reduction of both fibrosis and HCC [152] in a diethyl-nitrosamine (DEN)/CCl4 model of HCC with underlying fibrosis, that has been shown to closely mimic human disease [152,155].

Besides the major role of HSC in cirrhosis through collagen production, activated HSCs produce a plethora of growth factors, mitogens, cytokines and chemokines that induce tumor cell proliferation and migration and shape the tumor environment in favor of tumor growth. HSCs produce IL-6, IL-8 and Hepatocyte growth factor (HGF) that are known inducers of Heps proliferation [156]. Simultaneously, HSCs produce immune-regulatory cytokines, such as Monocyte Chemoattractant Protein-1 (MCP-1), attracting inflammatory cells [156]. Furthermore, activated HSCs are able to produce VEGFa and angiopoietins that act in a paracrine way on the adjacent liver sinusoidal endothelial cells (LSECs) to induce neovascularization [156]. Interestingly, LPA has been shown to induce the secretion of IL6, IL-8, HGF, MCP-1, regulated-on-activation-normal-T-cell-expressed-and-secreted (RANTES), Chemokine (C-C motif) ligand 21 (CCL21) and VEGFa in other cell types [157]. LPA stimulates expression of IL-6 in human dermal fibroblasts [158], induces IL-8 production in lymphatic endothelial cells [159], induces MCP-1 secretion in myotubes [160] and promotes VEGFa production [161]. Thus, it is possible that such LPA effects apply to activated HSCs as well, although this remains to be proven.

In conclusion, LPA is involved in the fibrotic process by positively modulating the activation of HSCs, a key cell type for collagen production and fibrosis progression. In turn, liver fibrosis creates a tumor-promoting microenvironment. Remaining questions concern the putative involvement of LPA in other tumor-promoting roles of HSCs, as well as the implicated LPARs. Furthermore, it is still unclear whether, apart from the evident increased hepatic LPA production by ATX, decreased LPA degradation by LPPs also contributes to the elevated LPA levels observed in cirrhotic patients or not.

### 7.2. Viral Hepatitis-Related HCC

Viral hepatitis is another risk factor for HCC with Hepatitis B virus (HBV) increasing HCC risk by 50–100 fold and Hepatitis C virus (HCV) by 20 fold [162]. Even though incorporation of the HBV virus [163] and possibly the HCV [164] in the DNA of Heps induces several mutations that might be responsible for Heps malignant transformation [165], HCC is mostly induced in cirrhotic chronic viral hepatitis (CVH) patients than in non-cirrhotic ones, suggesting that signals from the cirrhotic microenvironment are required to support growth of the transformed Heps. Increased levels of serum ATX activity and protein levels have been found in both patients with chronic hepatitis C (CHC) [152,166,167] and chronic hepatitis B (CHB) [168], while ATX activity strongly correlated with LPA levels [166] in CHC patients. Apart from the circulation, ATX was also found increased in the liver of CHC patients [169]. A probable cause of liver-specific ATX expression in CHC is the direct induction of ATX gene expression by HCV itself, as shown in vitro in HuH7 cells infected with HCV or in vivo in mice having chimeric livers with human Heps infected with HCV [170]. Notably, the HCV infection induces ATX expression in human Heps in vivo in the absence of any inflammatory response [170]. In line with this, in CHC patients with sustained anti-viral response [171] plasma ATX levels were decreased. Besides HCV infection, inflammatory, fibrotic or necrosis-associated factors cannot be excluded as possible ATX stimuli, since both viral and necro-inflammatory activities were reduced in parallel with systemic ATX levels reduction [171]. Indeed, hypoxic conditions that exist in CHC and other chronic liver diseases with high necro-inflammatory activity can also induce ATX [170], whereas ATX expression correlates with advanced HCV disease characterized by advanced stage of fibrosis [172]. Furthermore, inflammatory mediators such as TNF-a were also shown to induce ATX expression in primary Heps [152] and hepatoma cell lines [150].

Again, a causal relationship between ATX and HCV infection seems to be reciprocal, i.e., ATX may promote CHC. Genetic deletion or pharmacological inhibition of ATX reduced HCV proliferation, without affecting the ability of HCV to infect Heps, whereas administration of LPA had the opposite effect. The underlying mechanism of these effects involved stabilization of HIF-1a from ATX through LPA/LPAR1,3 which is important for sustaining HCV proliferation [170]. Furthermore, ATX may be also causally linked to immune-activation during HCV infection and HCV-HIV co-infection [173].

Apart from CVH, transcriptomic and data mining analysis showed a significant increase of ATX in HCV- and HBV-associated HCC compared to healthy controls [152,170]. Similarly, ATX was found significantly elevated in hepatitis-related HCC tissues, compared to tissues that developed HCC on a “non-inflammatory” background [150], suggesting that ATX upregulation in HCC requires the presence of an inflammatory and fibrotic component that exists in CVH. A pairwise analysis of tumor and non-tumor tissue from a big Chinese cohort of CHB patients with HCC showed that ATX is significantly upregulated in tumor compared to non-tumor tissue and the same applies to LPAR6 [174]. Hypoxia is a putative stimulus of the increased ATX since the hypoxia score is increased in HCC tumor tissue compared to adjacent non-tumor tissue and hypoxia has been shown, in the same study, to directly induce ATX expression in liver sections [170]. Moreover, ATX mRNA expression positively correlates with the hypoxia gene signature. The increased levels of ATX in the tumor leads to LPA production that has been shown to induce invasiveness of Hep3B cells, an HCC cell line with integrated the viral genome of HBV [150]. Furthermore, LPA through stabilization of HIF-1a (a master regulator of the glycolysis in tumors) [120] may alter cellular metabolism of HCV or HBV-infected Heps towards glycolysis, a key metabolic pathway that fuels cancer and stromal cells as shown in ovarian cancer [118,119].

Summing up, ATX induced by HCV infection, liver damage, chronic inflammation or hypoxia appears to be associated with the predisposition of CVH patients to develop HCC through HIF-1a stabilization. However, in vivo studies addressing the role of ATX/LPA in HCV/HBV-driven HCC are lacking due to the absence of animal models that would support HCC growth in HCV/HBV-infected mice with humanized livers. Such studies would be very informative about the potential use of ATX or LPARs as chemoprevention targets for the development of HCC in CVH patients.

### 7.3. Metabolic Diseases-Related HCC

In western countries, NAFLD is the second major cause of cirrhosis with HCC after alcohol [130] although its increasing incidence may render it first in the future. NAFLD can be histologically subdivided into simple steatosis and non-alcoholic steatohepatitis (NASH), which includes steatosis, lobular inflammation and fibrosis [175]. Of note, the increased incidence of NAFLD in the last decade was associated with the increased incidence of HCC in both cirrhotic and non-cirrhotic patients with NAFLD [176,177,178,179], thus challenging the concept that HCC develops mainly within liver cirrhosis. The increased appearance of HCC in non-cirrhotic NAFLD patients is probably οwed to the coexistence of NAFLD with obesity and T2DM. This coexistence increases the risk of HCC up to 25% [179], even in the absence of liver cirrhosis [180]. Obesity and T2DM are two fast-growing epidemics in industrialized countries, with dietary habits and sedentary lifestyle being their major risk factors, and are characterized by increased morbidity and mortality [181,182]. Besides, T2DM and obesity often coexist, thereby increasing the risk for HCC and CCA by two to three times [183] in all patients with chronic liver damage, independently of the etiology (viral, alcohol, metabolic), as well as mortality [184]. Moreover, HCC is now the leading cause of obesity-related cancer deaths in middle-aged men in the USA [179]. These observations suggest that metabolic alterations in the liver due to obesity or T2DM support a tumor-promoting microenvironment. In spite of the advances in the identification of the pathways involved in NAFLD progression in the presence of T2DM and obesity, the molecular pathways linking NAFLD and its risk factors (T2DM, obesity) with liver cancer remain poorly understood.

As mentioned above, adipose tissue expresses ATX abundantly (38–50%of plasma LPA derives from adipose-tissue ATX [26,27]) which is important for visceral and subcutaneous adipogenesis since adipose-specific ATX deletion reduces the adipocyte size upon high fat diet and global ATX deletion in adult mice reduces the adipocyte size in chow and high fat diet-fed animals in both fat depots [68]. Apart from adiposity, ATX/LPA also affect lipid metabolism. Lipid metabolism is an integral part of the hepatic function interconnected with glucose metabolism [185]. Accordingly, alterations in either glucose or free fatty acid levels in the context of T2DM, obesity and NAFLD may alter the metabolic activity of Heps possibly favoring their malignant transformation. Such a metabolic alteration appears to be the deregulated phospholipid metabolism that leads to an increased amount of LPA as evidenced by the observed increased ATX and LPA levels in the circulation of obese, T2DM and NAFLD patients [27,148,186,187,188,189]. Free fatty acids levels, high levels of glucose and the increased number of adipocytes are probably responsible for the increased ATX and LPA circulatory levels observed in NAFLD coexisting with T2DM and obesity. In line with this, blood ATX and LPA levels are also independently associated with liver steatosis and insulin resistance [187,188] as well as liver fibrosis severity [148]. Furthermore, in older or obese people, serum ATX correlates with multiple measures of adiposity and glucose homeostasis / insulin action [188]. Moreover, hepatic ATX expression was also found increased in patients with NAFLD as shown by data mining analysis [152]. A possible cause for that increase in the liver could be the increased LPC levels in NAFLD patients [190], given that LPC was found to be a potent stimulus of ATX in Heps [152]. However, studies examining the ATX/LPARs/LPPs mRNA expression or LPA levels in NAFLD-related HCC are lacking.

Insulin resistance, a key pathogenic event that drives T2DM [191,192,193,194] is driven by ectopic lipid deposition in liver (NAFLD) and muscle [195,196]. Ectopic lipid accumulation is the result of lipolysis taking place in the adipose tissue due to adipose tissue inflammation triggered by chronic low-grade inflammation in obese T2DM patients. Of note, alterations in the liver lipid/glucose metabolism and liver mitochondrial function also drive the appearance of fatty liver and, subsequently, insulin resistance. The latter further exacerbates fatty acid oversupply by increasing de novo hepatic lipogenesis [193,196,197] forming a vicious circle. ATX was shown to be involved in all the etiopathogenic mechanisms of insulin resistance including liver steatosis, adiposity, adipose tissue inflammation and impaired mitochondrial function as discussed below.

Adipose tissue-specific deletion of *Enpp2* in HFD-fed mice decreased adipose tissue inflammation locally [68] and liver steatosis, endocrinically, phenocopying the inducible global ATX deletion in adult mice [68]. Therefore, increased ATX levels in NAFLD may induce liver steatosis likely through increased adipose tissue inflammation and lipolysis. Furthermore, haploinsufficiency or induced, complete deletion of *Enpp2* improves glucose tolerance and insulin sensitivity in HFD-fed mice [68,198]. A possible LPAR through which LPA mediates this effect is LPAR1 since the *Lpar1*-knock out KO is also resistant to diet-induced obesity as is the *Enpp2*-KO [199]. The underlying mechanism for this effect involves an LPA-mediated decrease in insulin-stimulated AKT phosphorylation in white adipose tissue, liver, heart, and skeletal muscle and in mitochondrial energy metabolism in myotubes [198]. LPA has also been shown to induce glycogenolysis in Heps in vitro [200], to inhibit insulin-mediated glycogen synthesis in Heps [189], to inhibit glucose-induced insulin secretion from isolated pancreatic β islets in vitro while LPA injection has been shown to worsen glucose tolerance in vivo [201]. On the other hand, according to another study LPA enhanced the uptake of glucose in adipocytes by inducing the translocation of Glucose transporter 4 (GLUT-4) to the plasma membrane and lowered blood glucose in mice [202]. Besides, LPA was shown to promote a glycolytic shift in ovarian cancer cells [118] although it remains to be examined whether LPA can have the same effect in liver cancer cells. Therefore, LPA seems to affect glucose metabolism at several stages (absorption by the cell, release from the glycogen storage and oxidation for energy production) and can be envisioned as a lipokine, a term coined to describe a lipid hormone responsible for linking adipose tissue to systemic metabolism [203], as has already been proposed [204].

**Figure 3:**
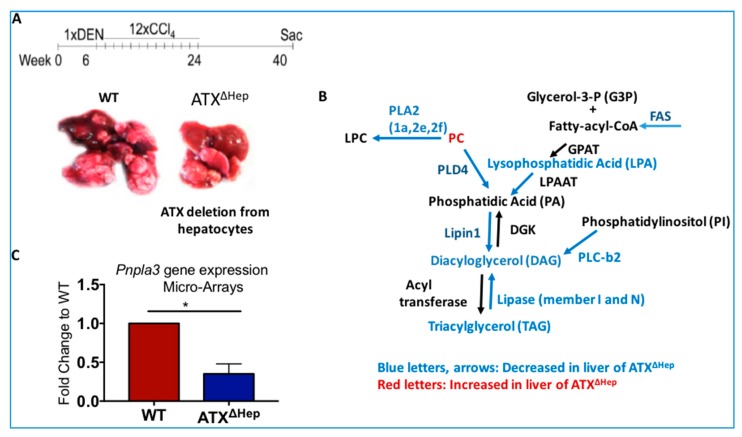
ATX genetic deletion from Hepatocytes (ATX^ΔHep^) diminishes (**A**) tumor growth and (**B,C**) gene expression of key enzymes involved in lipogenesis compared to littermate WT mice. DGK: Diacylglycerol kinase; FAS: Fatty Acid Synthase; GPAT: Glycerol-3-phosphate acyltransferase; LPAAT: Lysophosphatidic acid acyltransferase; PLA2: Phospholipase A2; PLC: Phospholipase C; PLD: Phospholipase D. Results are from [152].

It is well known that liver lipid synthesis is balanced with liver lipid export and lipid degradation which transforms lipids in small metabolites unable to be stored. Under chronic liver damage of viral, alcohol or metabolic cause, this homeostatic mechanism is disrupted leading to abnormal lipid accumulation in the form of lipid droplets in the liver with the highest degree existing in the alcoholic and non-alcoholic steatohepatitis [205]. In this context, Heps-derived ATX/LPA are also involved in neutral lipid liver deposition since Hep-specific deletion of *Enpp2* reduces liver lipid deposition in both toxin-induced chronic liver fibrosis and HCC animal models [152]. Abnormal lipid accumulation is observed in many HCC types and may serve as an energy source that supports cancer cell growth and proliferation [206]. Indeed, transformation of carbohydrate to fatty acids that are then esterified to triacylglycerols (TAGs), creates an energy storage that could provide energy, via β-oxidation, upon increased energy demands. This pathway is called *de novo* lipogenesis (DNL) and primarily takes place in the liver and adipose tissue. Deregulations in the lipogenic pathway are associated with diverse pathological conditions and cancer [207]. The increased endogenous lipid synthesis is primarily mediated by overexpression of fatty acid synthase (FAS), acetyl-CoA carboxylase (ACC) and Stearyl-CoA desaturases (Scds). Changes in these key lipogenic enzymes are critical for the development and maintenance of the malignant phenotype. Microarray and lipidomic analysis in the liver tumors shows alterations in lipid related genes and in lipid metabolism respectively, upon Hep-specific ATX deletion. Specifically, mice with Hep-specific ATX deletion at the HCC stage have reduced expression of several enzymes that regulate DNL compared to their littermate wild type (WT) controls (Figure 3), however, the mechanism that leads to these alterations remains unknown. In line with this, treatment of human hepatoma cell lines with LPA directly promotes TAG deposition and induces SCD, an enzyme involved in unsaturated fatty acid synthesis in humans. Apart from SCD, ATX/LPA may also affect other enzymes of the DNL pathway (Figure 3), as has been shown in the context of ovarian cancer [208].

Of note, patatin-like phospholipase domain-containing protein 3 (*PNPLA3*), a lipase that strongly associates with NAFLD progression to NASH and HCC [209], was also found downregulated in the liver of Hep-specific ATX deletion (ATXΔHep) mice (Figure 3B). Interestingly, PNPLA3 rs738409 C>G polymorphism may increase the HCC risk in the virus and alcohol-related HCC in caucasians [210], as does for NAFLD related HCC [211]. However, it remains to be examined whether ATX through LPA directly affects PNPLA3 function. Nonetheless, these observations suggest that LPA is involved in the altered lipid metabolism which favors malignant transformation of Heps across HCC of different causes.

Conclusively, these data suggest that LPA directly induces adipogenesis, glycogenolysis, glycolysis, adipose tissue inflammation and adipose tissue lipolysis and in parallel impairs skeletal muscle insulin signaling and mitochondrial function. These processes lead to liver steatosis, insulin resistance, glucose intolerance and lipogenesis. Lipogenesis fueled by insulin resistance is a pathogenic factor for NAFLD development [212] and is required for HCC growth [206]. Therefore, adipose tissue-derived ATX in obese people may be the cause of their increased susceptibility to T2DM and NAFLD and LPA may be involved in both fatty liver deposition and its progression to NASH. Moreover, ATX and LPA may exert their pathogenic role in HCC by altering tumor DNL and probably by fueling cancer cells with glucose. However, it is unknown whether the LPA effects on liver lipogenesis and glucose metabolism in HCC are direct or indirect and which are the signaling pathways that mediate these alterations. Moreover, it is still unknown whether the LPA-dependent effect of ATX on lipid metabolism is owed to extracellular LPA and its signaling through LPARs or whether the extracellular LPA is absorbed by the cells and subsequently serves as an intracellular intermediate for diacylglycerol/triacylglycerol (DAG/TAG) synthesis. Future studies employing tracking of labeled LPA could shed some light regarding these questions.

## 8. Deregulated LPA Signaling and HCC

Many studies have found increased LPA levels in HCC patients compared to patients with non-malignant liver disease [213]. Notably, targeted phospholipid analysis in human HCC specimens revealed a significant increase of choline (the byproduct of LPA generation) in tumor tissue, an increase of LPC within the bile whereas LPA was found increased in both tumor tissue and bile and additionally in serum [213]. Therefore, LPA is the only lipid of the LPC/ATX/LPA axis that remains increased in all the possible tissues/fluids in HCC, indicating either alternative ATX-independent pathways of LPA production or diminished degradation of the produced LPA, leading to a prolonged exposure of the cancerous liver to an increased amount of LPA. Differences in LPA species profile upon HCC occurrence also exist. The ratio of serum LPA species 20:4/18:2 was markedly increased in patients with HCC compared with patients with liver disease but no HCC, irrespective of the underlying etiology (hepatitis C, NASH or alcohol) [214]. Furthermore, in a seminal longitudinal study which attempted to identify a gene signature associated with high-risk for HCC (using transcriptome meta-analysis) in patients suffering from cirrhosis of all major etiologies (hepatitis B/C, alcohol, and NASH), LPA pathway activation was robustly observed in patients with high HCC risk [155]. The same applies for HCC rodent models. Therefore, LPA signaling has been proposed as a good target for chemoprevention in the context of HCC [155].

Strong evidence for the involvement of LPA on HCC growth comes from genetic studies which delete the LPA producing enzyme (ATX) in hepatocytes. Upon Hep-specific deletion of *Enpp2* in a DEN/CCl4 HCC model there is less HCC tumor growth compared to control mice [152]. Microarray analysis in these livers shows that among the different LPARs, only LPAR2 is downregulated upon ATX deletion from the liver tissue suggesting that LPAR2 may be responsible for the tumor-promoting role of LPA in that model [152]. However, in vivo studies addressing the genetic role of LPARs in HCC are lacking. Even though Hep-specific deletion of *Enpp2* reduces effectively the liver ATX/LPA levels in the fibrotic phase of the CCl4/DEN model (>95% reduction for both), this reduction is milder in the HCC phase (40% reduction for LPA, 80% reduction for ATX) [152] suggesting that even a 40% reduction of LPA is sufficient to significantly reduce tumor growth. This results also suggest that total LPA levels in liver cancer are not solely determined by hepatic ATX. An amount of LPA could derive from extrahepatic ATX or, as discussed above, from ATX-independent routes of LPA synthesis, such as LPA generation from phosphatidic acid (PA) [1] or from glycerol-phosphate through GPAT enzymes [215]. However, the most probable scenario is that the LPA levels are affected by decreased LPA degradation from LPPs (encoded by *Ppap2* genes). Indeed, data mining analysis in HCC tissue from patients and gene expression analysis in the HCC specimens from animal models show reduced expression of *Ppa2b* (the gene encoding LPP3) in the cancerous liver, suggesting that the half-life of local LPA in the malignant tissue is longer than in the non-malignant. Given the strong carcinogenic role of LPA across different types of cancer as discussed above (Section 5) and the plethora of enzymes that determine the total amount of LPA (LPPs, PLA1, GPAT), it remains to be examined whether simultaneous targeting of ATX and LPARs or LPPs is more effective than single targeting of ATX.

According to a study, LPAR1, 3 and 6 expression is increased in the livers of patients with HCC, and underlying cirrhosis or HCV infection, compared to healthy livers with LPAR6 expression being stronger in HCC tissue than in paired non-tumor liver tissue [174]. Data mining analysis has shown that even though the mRNA levels of all LPARs (LPAR1–6) and ATX are increased in the liver upon chronic liver diseases of different etiology (viral, alcohol, metabolic) and in the stage of liver cirrhosis, in the context of HCC only ATX, LPAR2 and LPAR6 are increased in the liver tumor compared to non-tumoral areas [152]. Interestingly, the rest of the LPARs (LPAR1, LPAR3–5) are decreased in the HCC liver as compared to adjacent normal tissue, suggesting that LPAR2 and LPAR6 are probably expressed in the tumor whereas the rest LPARs are expressed in the surrounding microenvironment. This is in accordance with a study showing that only LPAR2 and LPAR6 strongly associate with poor survival in HCC [216]. In addition, LPAR2 associates with T2DM [217] suggesting that ATX/LPA through LPAR2 may be involved in NAFLD severity and its progression to HCC in a diabetic background. RNAi-mediated genetic deletion of *Lpar6* impaired HCC tumor growth in tumor xenograft assays, probably through a Signal-transducer-and-activator-of-transcription-3 (STAT3)/proto-oncogene pim-3-dependent mechanism. Furthermore, expression analysis of LPAR6 in HCC tissue, shows a connection between LPAR6/Pim-3, high proliferation rates, and poorer survival outcomes [218]. These results suggest that LPAR6 may be important in HCC growth and progression. Regarding LPAR2, there are no further experimental studies addressing its role in HCC.

On the other hand, the rest of the LPARs (LPAR1, LPAR3 and LPAR5) presumably are involved in the tumor growth via modulating its microenvironment. Studies detecting the expression of LPARs in the liver show that during chronic liver damage LPARs are expressed by NPCs [153] whereas ATX is expressed by Heps [152] as discussed above and as shown in a rat model of liver cancer (low-dose of DEN) [155]. In the latter model, plasma ATX activity and LPAR1 expression in the liver increased as cirrhosis developed and while LPAR1 was mostly expressed in stellate cells, ATX was mostly expressed in Heps implying a crosstalk between the two cell types leading to the stimulation of LPA signaling [155]. In line with this, elevated LPAR1/LPAR3 expression in HCC was evidenced in the microenvironment between the tumor and non-tumor liver (NTL) and LPAR3 expression coincided with cancer stem cell markers expression [219]. However, other studies have pointed out also a role of LPAR1 in tumor *per se*. LPAR1 has been found expressed by Heps in human HCC [220]. LPAR1 expression correlated with EMT in tumor area and with the worse prognosis after liver resection. Furthermore, LPAR1 overexpression in HCC cells increased the EMT features including upregulation of Vimentin, Fibronectin and N-cadherin and suppression of E-cadherin [221]. In accordance with these, in vivo experiments with HCC xenografts driven from Huh7 cells encoding for LPAR1 showed that LPAR1 overexpression accelerates HCC cell growth on a nude mouse model [221]. The underlying mechanism of LPARs effects in the above studies was shown by in vitro assays where hepatoma cell lines were treated with LPA. The latter was shown to induce migration and invasion of hepatoma cells through Rho [222]. LPAR1 was shown to increase cell migration, invasion, cell viability and proliferation of HCC cells through PI3K/AKT/mTOR/Skp2/p27^Kip1^ signaling [220]. A cell population in the microenvironment between the tumor and the non-tumor liver, distinct from the tumor mass per se, and expressing LPAR3 and cancer stem cell markers without hepatocyte markers [219] was suggested to mediate tumor invasiveness through a G_αi_-ERK pathway. [219]. Furthermore, LPAR6 mediated HCC tumorigenicity in tumor xenografts, probably through a STAT3/pim-3-dependent mechanism [218]. LPA was also shown to induce mitochondrial dysfunction in myotubes [198]. Mitochondrial dysfunction, defined as a reduced ATP production by oxidative phosphorylation [193,197], can shift cell metabolism towards ATP generation by glycolysis (Warburg effect) [223] promoting malignant transformation of benign cells, and is associated with a more invasive and chemoresistant cancer phenotype [224]. Mitochondrial dysfunction has also been reported to be a cause of fibrosis [197,225] and inflammation [226,227], attributes that also characterize the tumor stroma.

Summing up, LPA produced extracellularly by ATX, has diverse biological activities implicated in tumor initiation and progression, including increasing cell survival, angiogenesis, lipid metabolism, glucose metabolism, mitochondrial function, invasion and metastasis (as explicitly discussed above). Even though LPA and LPARs are found increased by many studies in patients with HCC compared to patients with non-malignant liver disease, the role of LPARs in hepatic tumor pathology is hitherto poorly understood since in vivo studies addressing the role of LPARs in chronic liver diseases and HCC are lacking.

## 9. Deregulated LPA Metabolism and CCA

Most risk factors for CCA cause chronic inflammation and cholestasis [228]. LPA is linked to cholestasis as several LPA species are higher in the sera of women with intrahepatic cholestasis of pregnancy compared to matched healthy pregnant women [229] and intradermal injections of LPA in mice are able to induce pruritus [229]. Additionally, serum ATX activity and protein levels were also found increased in patients with cholestatic disorders and pruritus [229,230] and correlated with itch intensity [229,231]. Enteroendocrine cells of the small intestine might present an important source of cholestasis-induced serum ATX activity in humans, albeit not in mice [232]. From a mechanistic point of view, LPA has been shown to activate Yes-Associated-Protein (YAP) () [233], an oncogene that promotes CCA tumorigenesis in in vivo studies [234,235] and correlates with metastasis and poor prognosis in CCA patients [236].

CCA-associated lymphangiogenesis is significantly associated with increased lymphatic metastasis, recurrence of the tumor, and reduced overall survival in patients with CCA.LPA has been shown to induce the expression of vascular endothelial growth factor-C (VEGF-C), a lymphangiogenic factor, through LPAR1 and LPAR3 in prostate cancer (PCa) cells [237]. Thereby, LPA may also reinforce these features of CCA. However, in vivo mouse studies addressing the effect of ATX/LPA in the CCA growth are lacking as well as studies in patients with CCA.

Further insight in CCA is gained by the usage of multidrug resistance protein 2 (*Mdr2*) knockout mice which develop spontaneous cholestatic liver injury and fibrosis mirroring human PSC (a risk factor of CCA) through altered PC levels in the bile [238]. Deletion of *Mdr2* in Heps leads to reduced phospholipid secretion in the bile, affecting its chemical composition with an increased concentration of non-micellar-bound free bile acids, which exert a mutagenic effect on the Heps or cholangiocytes. Indeed, *Mdr2*-KO mice develop pre-neoplastic lesions and HCC at 4–6 months and at 12 months of age, respectively, suggesting that alteration in bile acids may induce mutagenesis and transformation of Heps towards to a malignant phenotype [239]. LPA was found increased in the circulation of *Mdr2* KO mice with its levels increasing significantly at the transition from a premalignant to a malignant phenotype, while combined pharmacological inhibition of ATX and LPAR1 reduced HCC growth [239]. Even though mice of that background do not develop CCA spontaneously, the reduction of HCC upon inhibition of LPA signaling suggests that LPA may be also involved in the connection between altered bile acid pool and malignant transformation of liver epithelial cells. Besides, in humans in the context of cholestasis, increased exposure of cholangiocytes to specific bile acids has been suggested to predispose them to CCA [240].

## 10. Pharmacological Targeting of HCC and Its Risk Factors

Pharmacological studies with chemical inhibitors that inhibit ATX activity and LPA signaling in the liver of NAFLD animal models show an effect only on liver fibrosis and not on liver steatosis [241], although a genetic confirmation of this effect is still lacking. Similarly, pharmacological inhibition of ATX in a CCl4 liver fibrosis model resulted in decreased histological fibrosis but no alteration in liver lipid deposition, even though genetic deletion of *Enpp2* in the same model ameliorates both liver lipid deposition and liver fibrosis. Regarding HCC, pharmacological inhibition of ATX resulted in decreased histological fibrosis and reduced HCC development in a DEN model of hepatic fibrosis and HCC, that has been shown to closely resemble human disease [152,155] and in a mouse model of PSC which also develops HCC [216,239]. In line with this, pharmacological inhibition of the LPA signaling with an inhibitor of LPAR1, AM095, reduced tumors in vivo, suppressed HCC high-risk genes and restored HCC low-risk genes in organotypic ex vivo culture of patient-derived fibrotic liver tissues [155]. Therefore, in vivo studies with chemical inhibitors confirm the genetic studies and provide proof of concept that the ATX/LPA axis is an interesting therapeutic target against both liver fibrosis and liver cancer. However, the differences between the outcomes of ATX pharmacological inhibition and genetic deletion of *Enpp2* need to be further investigated (pharmacological inhibition does not affect lipid metabolism and deposition as does genetic deletion). Presumably, these differences are owed to limited accessibility of the steatotic Heps or the adipocytes by the tested inhibitors, which could be tested by tissue imaging or the usage of other inhibitor formulations. The fact that the ATX/LPA axis can both delay liver fibrosis and prevent liver cancer is extremely important given that HCC is usually developed in the background of liver fibrosis. Indeed, therapies that can both retard the progression of fibrosis and the development of HCC would be very promising for liver disease patients. Interestingly, inhibitors of the ATX/LPA axis are already being tested in clinical trials. A promising ATX inhibitor, GLPG1690 from Galapagos, is currently in phase 3 clinical trials against IPF. (NCT03711162; NCT03733444). An LPAR1 antagonist, BMS-986020 from Bristol Myers Squibb, is in phase 2 clinical trials, (NCT01766817) against IPF too, although this inhibitor has caused an increase in hepatic enzymes [78]. Moreover, SAR100842, another LPAR1 antagonist, is in phase 2 clinical trials for the treatment of systemic sclerosis (NCT01651143) [242] and an antibody against LPA, Lpathomab, is in phase 1a. (NCT02341508).

## 11. Conclusions

**Figure 4:**
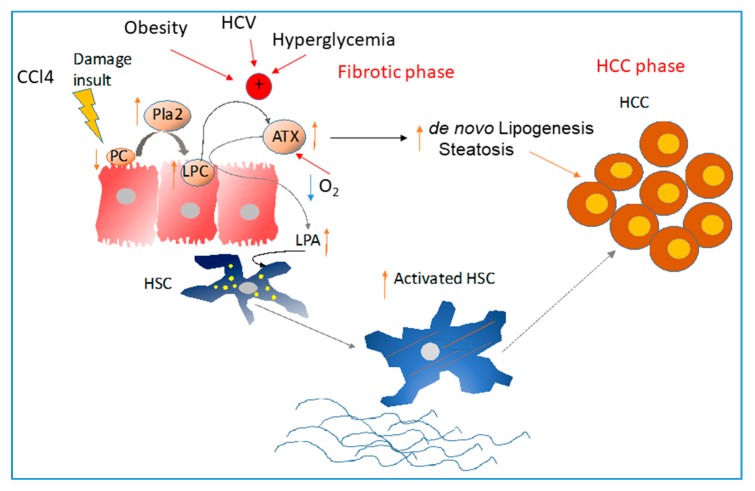
Hepatocyte specific ATX production driven by fat, glucose, phospholipids and Hepatitis C virus (HCV) infection can promote hepatic stellate cells (HSCs) activation, steatosis and hepatocellular carcinoma (HCC) growth.

Liver cancer is one of the leading causes of death worldwide in men. NAFLD coexisting with obesity and diabetes mellitus are new emerging risk factors of liver cancer in westernized countries. Since diabetes and obesity incidence is increasing dramatically in the last years, there is an urgent, but still unmet, need to identify suitable targets that link metabolic diseases with liver cancer. Therapies that can delay the progression of fibrosis to cirrhosis, restore the distorted liver metabolism and prevent the development of liver cancer would be very promising. The ATX-LPA pathway was identified as a regulator of HCC risk in human cirrhosis patients and appears to be an appealing pharmacological target since genetic deletion of *Enpp2* ameliorates liver fibrosis, liver steatosis, insulin resistance, glucose intolerance, visceral and subcutaneous adiposity and reduces HCC development in animal models (Figure 4). In line with this, in the context of HCC ATX/LPA seem to be involved in the energy reprogramming/lipid metabolism of cancer cells. Finally, common stimuli that drive liver damage (obesity, fat, glucose, HCV virus) can also activate ATX (Figure 4) suggesting that ATX is linked to both the etiopathogenesis and the progression of liver diseases. LPA metabolism and signaling encompass enzymes involved in LPA production (ATX), LPA degradation (LPPs) and LPARs that transmit the LPA signal. However, the sole genetic evidence for the role of this pathway in liver cancer concerns ATX. Furthermore, in vivo genetic exploration of the role of the remaining components of the LPA axis (LPPs, LPARs) in liver pathology/HCC is expected to reveal new therapeutic targets. Combinatorial targeting of ATX and a second molecule of the LPA axis is likely to be effective for the prevention or treatment of liver cancer in patients with chronic liver diseases.
